# Genome-Wide Identification of Mango (*Mangifera indica* L.) Polygalacturonases: Expression Analysis of Family Members and Total Enzyme Activity During Fruit Ripening

**DOI:** 10.3389/fpls.2019.00969

**Published:** 2019-07-30

**Authors:** Mitzuko Dautt-Castro, Andrés G. López-Virgen, Adrian Ochoa-Leyva, Carmen A. Contreras-Vergara, Ana P. Sortillón-Sortillón, Miguel A. Martínez-Téllez, Gustavo A. González-Aguilar, J. Sergio Casas-Flores, Adriana Sañudo-Barajas, David N. Kuhn, Maria A. Islas-Osuna

**Affiliations:** ^1^Laboratorio de Genética y Biología Molecular de Plantas, Centro de Investigación en Alimentación y Desarrollo, A.C. (CIAD), Hermosillo, Mexico; ^2^Laboratorio de Genómica Funcional y Comparativa, División de Biología Molecular, IPICYT, San Luis Potosí, Mexico; ^3^Departamento de Microbiología Molecular, Instituto de Biotecnología, Universidad Nacional Autónoma de México (UNAM), Cuernavaca, Mexico; ^4^Laboratorio de Bioquímica, Centro de Investigación en Alimentación y Desarrollo, A.C. (CIAD), Unidad Culiacán, Culiacán, Mexico; ^5^Agricultural Research Service, Subtropical Horticulture Research Station, United States Department of Agriculture, Miami, FL, United States

**Keywords:** polygalacturonase, *Mangifera indica* L., gene expression, enzymatic activity, firmness, ripening

## Abstract

Mango (*Mangifera indica L*.) is an important commercial fruit that shows a noticeable loss of firmness during ripening. Polygalacturonase (PG, E.C. 3.2.1.15) is a crucial enzyme for cell wall loosening during fruit ripening since it solubilizes pectin and its activity correlates with fruit softening. Mango PGs were mapped to a genome draft using seventeen PGs found in mango transcriptomes and 48 bonafide PGs were identified. The phylogenetic analysis suggests that they are related to *Citrus sinensis*, which may indicate a recent evolutive divergence and related functions with orthologs in the tree. Gene expression analysis for nine PGs showed differential expression for them during post-harvest fruit ripening, *MiPG21-1*, *MiPG14*, *MiPG69-1*, *MiPG17, MiPG49*, *MiPG23-3*, *MiPG22-7*, and *MiPG16* were highly up-regulated. PG enzymatic activity also increased during maturation and these results correlate with the loss of firmness observed in mango during post-harvest ripening, between the ethylene production burst and the climacteric peak. The analysis of PGs promoter regions identified regulatory sequences associated to ripening such as MADS-box, ethylene regulation like ethylene insensitive 3 (EIN3) factors, APETALA2-like and ethylene response element factors. During mango fruit ripening the action of at least these nine PGs contribute to softening, and their expression is regulated at the transcriptional level. The prediction of the tridimensional structure of some PGs showed a conserved parallel beta-helical fold related to polysaccharide hydrolysis and a modular architecture, where exons correspond to structural elements. Further biotechnological approaches could target specific softening-related PGs to extend mango post-harvest shelf life.

## Introduction

Fruit ripening is a physiological, biochemical, and genetically programmed process. This process is characterized by rheological and texture changes as the cell wall is disassembled by the action of hydrolytic enzymes (Li et al., 2010). Ripening leads to desirable sensorial characteristics of the fruit besides softening, such as aroma and color development (White, 2002). Accelerated mesocarp softening that characterizes mango fruit ripening occurs at the climacteric peak (Litz, 2009). The cell wall is a scaffold made of complex polysaccharides (pectins, cellulose, hemicelluloses, among others) that during ripening are hydrolyzed by enzymes such as polygalacturonases (PGs), pectate lyases, β-galactosidases, xylanases, glucosidases, among others (Brummell and Harpster, 2001; Brummell, 2006). Genetic modifications like suppression or over-expression of genes that encode for these hydrolytic enzymes have provided information about their function as well as the redundancy in the function of several isoforms that participate during fruit ripening (Goulao and Oliveira, 2008). Recently, mango mesocarp transcriptomes have been obtained (Dautt-Castro et al., 2015, 2018); thus family members encoding these hydrolases can be identified and further gene expression studies performed to understand more about their roles in the quick softening of mango fruit.

Polygalacturonases are cell wall disassembling enzymes with profound influence in fleshy fruit softening during ripening. Their specific function is pectin degradation, which is a structural polysaccharide of the primary cell wall and middle lamella, composed mainly of α-1, 4 D-galacturonic acid sugars (Lang and Dörnenburg, 2000). Several PG genes have been identified in fruits like banana (*Musa accuminata*) (Asif and Nath, 2005), fleshy fruit of oil palm (Roongsattham et al., 2012), papaya (*Carica papaya*) (Fabi et al., 2014), cucumber (*Cucumis sativus*) (Yu et al., 2014), among others. Also, transcriptomic analysis of nectarine (*Prunus persica* L.), orange (*Citrus sinensis*) and melon (*Cucumis melo* L.), among others, have revealed PG genes associated to fruit ripening (Ziliotto et al., 2008; Corbacho et al., 2013; Yu et al., 2014). Recently, with the sequencing of genomes from different plants, it has been possible to uncover whole families of PGs. Ke et al. (2018), identified and characterized 54 PGs in tomato, which were classified into seven clades. These clades have been related to specific functions and tissues in plants, for example, members from clades A and B have a role in fruit and abscission zone development, while clades C, D, and F members are involved in flowering development (Liang et al., 2015).

Efforts have been made to understand more about mango softening and few reports have focused on PG activity under different ripening stages or treatments. For example, PG isoforms have been identified in mango “Alphonso” (Prasanna et al., 2006), “Dasheari” (Singh and Dwivedi, 2008) and “Nam Dok Mai” (Suntornwat et al., 2000). In mango “Kent” and “Ataulfo” enzymatic activity of PG has been reported as well as the effect of the ethylene-antagonist 1-methyl cyclopropene (1-MCP) (Muy Rangel et al., 2009; Islas-Osuna et al., 2010). Only few gene expression studies were addressed using “next generation sequencing (NGS)” (Dautt-Castro et al., 2015, 2018) plus access to a mango genome draft from Tommy Atkins cultivar (Kuhn, personal communication). Therefore, the present study aimed to identify PG family members through transcriptomes and within the mango genome, to uncover their gene structure and phylogenetic relationship as well as to evaluate the expression of some *PGs* in mango mesocarp at different ripening stages, and PG enzymatic activity.

## Materials and Methods

### Plant Material

Mango (*Mangifera indica* L.) fruit cultivar “Kent” were hand-harvested in a commercial orchard located in Navojoa, Sonora, México (27°03′49.33″ N and 109°30′11.42″ W). Fruits were selected at physiological maturity stage according to fruit shape, peel color, size and at 125 days after anthesis. Mangos were transported to the laboratory where they were disinfected with chlorinated water and stored at 20°C with 60–65% humidity during 16 days.

### CO_2_, Ethylene Measurement

CO_2_ and ethylene production were measured by gas chromatography (Varian Star 3400, Varian United States) equipped with thermal conductivity (TCD) and flame ionization detectors (FID) and a 2 m × 1/82″ metal column filled with Hayesep N 800/100. These measurements were done after 24 h of harvesting mango. The fruit was placed in sealed plastic containers (2 L) for 2 h at 20°C, then CO_2_ and ethylene head-space concentration were analyzed by withdrawing 1 mL sample from the container and injecting them into the gas chromatograph (Muy Rangel et al., 2009). A standard of 5% of CO_2_ and 0.1% for ethylene was used. Gas concentrations were estimated using the following equations:

$$m\,l\,C\,O\,2/K\,g*h=\frac{\left(s\,a\,m\,p\,l\,e\,a\,r\,e\,a\right)\,\left(\frac{s\,t\,a\,n\,d\,a\,r\,d\,c\,o\,n\,c\,e\,n\,t\,r\,a\,t\,i\,o\,n}{s\,t\,a\,n\,d\,a\,r\,d\,a\,r\,e\,a}\right)\,\left(h\,e\,a\,d\,s\,p\,a\,c\,e\,a\,r\,e\,a\right)}{\left(i\,n\,c\,u\,b\,a\,t\,i\,o\,n\,t\,i\,m\,e\right)\,\left(s\,a\,m\,p\,l\,e\,w\,e\,i\,g\,h\,t\right)}$$

$$\mu \,l\,C\,2\,H\,4/K\,g*h=\frac{\left(s\,a\,m\,p\,l\,e\,a\,r\,e\,a\right)\,\left(\frac{s\,t\,a\,n\,d\,a\,r\,d\,c\,o\,n\,c\,e\,n\,t\,r\,a\,t\,i\,o\,n}{s\,t\,a\,n\,d\,a\,r\,d\,a\,r\,e\,a}\right)\,\left(h\,e\,a\,d\,s\,p\,a\,c\,e\,a\,r\,e\,a\right)}{\left(i\,n\,c\,u\,b\,a\,t\,i\,o\,n\,t\,i\,m\,e\right)\,\left(s\,a\,m\,p\,l\,e\,w\,e\,i\,g\,h\,t\right)}$$

### Fruit Firmness

Firmness was measured using a digital texturometer (Chatillon Model TCM200). Mango pulp firmness was measured in two sites of the fruit, and the average was reported (Cárdenas-Coronel et al., 2012). The loss of firmness was reported as newtons (N).

### Identification of Mango PG Family Genes in Transcriptome and Mapping to the Mango Genome

Two mango mesocarp transcriptomes (GenBank accessions PRJNA258477 and PRJNA286253) were used to identify candidate *PG* transcripts, and they were mapped into the mango cv. Tommy Atkins genome (DK, personal communication) to obtain information like the number of exons, and chromosomal localization of each gene. The deduced amino acid sequences were analyzed using the BLAST algorithm against the GenBank database. Also, other bioinformatics tools like gene ontology (GO) to know the biological process, molecular function and cellular component of the *PG* genes, as well as the clusters of orthologous groups (COG) to identify Endo and Exo PGs were used. To predict the theoretical molecular weight and the isoelectric point of the deduced PGs proteins, the compute pl/Mw tool of ExPASy^1^ was used. Multiple sequence alignments of the PGs sequences were done with CLUSTAL W, and the figures were made using BoxShade software^2^, to show the sequence similarity of enzymes within this family. The prediction of signal peptides in PG sequences was carried out using SignalP 5.0^3^.

### Phylogenetic, Gene Structure of PG Genes and *Cis*-Regulatory Elements Analysis

All 48 PG protein sequences from the mango genome were aligned along 69 PG from Arabidopsis *thaliana*, using the Neighbor-Joining method, with a previous alignment using the algorithm MUSCLE. The bootstrap consensus tree inferred from 2000 replicates is taken to represent the evolutionary relationship of the taxa analyzed. Based on the phylogenetic tree, mango PG sequences were named according to most nearby *A. thaliana* sequences. All phylogenetic inferences were conducted in MEGA (Kumar et al., 2016).

The full-length amino acid sequences of the PGs encoded in the mango genome were compared among them using multiple sequence alignments with MUSCLE and using the default settings (Edgar, 2004). The phylogenetic tree was constructed using the neighbor-joining method (Saitou and Nei, 1987) with a bootstrap value of 2000 replicates in MEGA (Kumar et al., 2016). To assign clades, a neighbor-joining tree was obtained comparing *Solanum lycopersicum* PG amino acid sequences with mango (*M. indica* L.) PGs. Trees were drawn using iTOL server (Letunic and Bork, 2016). The structure of the introns and exons of the *MiPG* genes were obtained using GSDS 2.0 (Hu et al., 2015)^4^.

A total of 1.5 Kb of genomic DNA sequences upstream of the initiation codon (ATG) of each *MiPG* gene was obtained from the mango genome database. These promoter sequences were used to investigate the regulatory elements in the MatInspector program, using general core promoter elements and plants matrix groups, with 0.90 of core and matrix similarity. Also, analysis of overrepresented transcription factor binding sites was carried out using genomic and promoter background of *A. thaliana*, TAIR 10 (Cartharius et al., 2005).

### RNA Isolation, cDNA Synthesis and Relative Expression of PGs by qRT-PCR

Total RNA was isolated from the mango mesocarp tissue according to López-Gómez and Gómez-Lim (1992), at days 1, 4, 7, 10, and 16 of post-harvest storage. The RNA quantity was estimated at 260 nm using a Nano-Drop ND-1000 UV-Vis spectrophotometer (Nano Drop Technologies Inc., Wilmington, DE, United States). RNA integrity was assessed using agarose gel electrophoresis under denaturing conditions. RNA was treated with RNA-free DNase I (Roche, CA, United States) to eliminate the genomic DNA. Then, the cDNA synthesis was performed by reverse transcription from 2.5 μg of total RNA, using the SuperScript II kit (Invitrogen, CA, United States) according to manufacturer conditions.

Quantitative PCR was carried out using iQ^TM^ SYBR^®^ Green Supermix (Bio-Rad, CA, United States). All samples were PCR-amplified by triplicates in reactions which included 100 ng of cDNA template, 12.5 μL of SYBR^®^ Green qRT-PCR Master Mix, 1 μL of 5 μM sense primer, 1 μL of 5 μM antisense primer and water to 25 μL of final volume. Specific primers to amplify the nine PG genes and the reference gene *GAPDH* (glyceraldehyde 3-phosphate dehydrogenase) are shown in Supplementary Table S1. The PCR products were amplified in a Step-One^TM^ Real-time PCR System (Applied Biosystems). Amplification conditions were one cycle of 95°C for 10 min and 40 cycles of 95°C for 15 s and 60°C for 1 min. PCR product specificity was confirmed by constructing a melt curve after amplification raising the temperature from 95°C for 15 s, 60°C for 1 min and 95°C for 15 s. Non-template controls were included during each gene amplification. The method 2^–ΔΔC^_T_ was used to evaluate changes in the relative mRNA amount of target genes (Livak and Schmittgen, 2001). The results are expressed as relative mRNA steady-state levels of the target gene and normalized to the *GAPDH* (Dautt-Castro et al., 2015) and *ACT 7* (Tafolla-Arellano et al., 2017) expression levels. The differential expression obtained by RNA-Seq was also used to compare the expression patterns of PG genes and qPCR was used to validate those.

### PG Enzymatic Activity

PG enzymatic activity was determined according to Gross (1982). Mesocarp tissue (25 g) was homogenized with 75 ml of 1% sodium bisulfite pH 6. The homogenized was filtered, and the residue was suspended in 75 ml of 1% sodium bisulfite (pH 6), filtered again and suspended in 37.5 ml of sodium chloride 1 M. The pH was adjusted at 6 with NaOH 1 N, and the solution was stirred for 3 h at 4°C. After agitation, the solution was filtrated and centrifuged at 9000 × *g* for 15 min. Aliquots of 2.5 ml of supernatant were desalted in a Sephadex G25 column equilibrated with sodium acetate 50 mM (pH 4). The enzymatic extract (50 μl) was incubated for 2 h at 30°C in a 0.2 ml solution containing sodium acetate 37.5 mM (pH 4.4) and 0.2% polygalacturonic acid previously washed with 80% ethanol. The reaction was stopped by addition of 1 ml of cold borate buffer 100 mM (pH 9). Then, 0.2 ml of 1% 2-cianoacetamide was added, and the samples were placed in boiling water by 10 min. Finally, the samples were cooled at room temperature and the absorbance was read at 276 nm. Protein was quantitated by the method of Bradford (1976). The results were expressed by nmol of reducing groups produced for mg of protein for an hour.

### Molecular Modeling of PGs

The amino acid sequences of MiPG14, MiPG21-1, MiPG23-3, MiPG49, MiPG46-3, and MiPG69-1 were modeled by using Phyre2 server (Kelley et al., 2015). The crystallographic model used as a template was the rhamnogalacturonase A from *Aspergillus aculeatus* (PDB 1RMG). Figures were build using PyMol (DeLano, 2002; Schrödinger, 2019) the structures were colored according to the color code used for exons in the sequence alignments.

### Statistical Analysis

Statistical analysis was performed using one-way ANOVA, with a 0.05 significance level (*P* < 0.05). Fisher’s test was used to detect statistical differences between means, using the XLSTAT software.

## Results and Discussion

### CO_2_ and Ethylene Levels Are Characteristic of a Climateric Fruit

**FIGURE 1:**
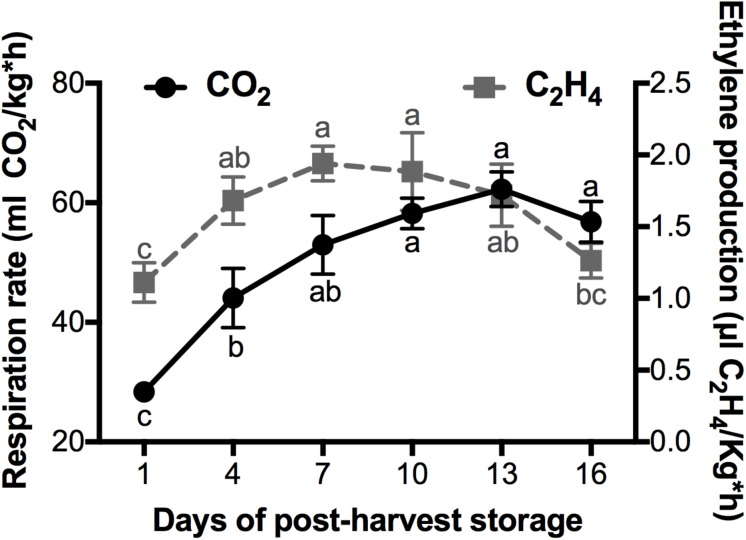
Evaluation of CO_2_ and Ethylene production (black circles and gray squares, respectively) of mango fruits cultivar “Kent” harvested at the stage of physiological maturity and stored at 20°C for 16 days at an RH of 60–65%. Data shown represent the average of five biological replicates (*n* = 5). Error bar indicates SE of the means and different letters indicate significant differences. Statistical significance between the control and treatment groups was determined by one-way ANOVA with the Tukey-Kramer test (*P* < 0.05).

Mango belongs to the climacteric fruits, in which the CO_2_ and ethylene present a peak of production during post-harvest (Bouzayen et al., 2010). The respiration rate and the ethylene production observed in mango cv. Kent fruits during their ripening at a storage temperature of 20°C is shown in Figure 1. The maximum ethylene production occurred at day 7 of post-harvest storage, with a value of 1.9 uL C_2_H_4_/Kg^*^h. This ethylene accumulation preceded the climacteric peak that occurred at day 13 with a CO_2_ production of 62.29 mL CO_2_/kg^*^h. It is well documented that ethylene production during climacteric ripening triggers signal transduction for the activation of several transcription factors that in turn activate the expression of genes that encode enzymes that catalyze ripening changes such as color, flavor, texture, aroma, among others (Grierson, 2013). In agreement with this, most of the results that we discuss later are related to the increase of ethylene production.

### Mango Suffered a Drastic Loss of Firmness by Day 10 of Post-harvest at 20°C

**FIGURE 2:**
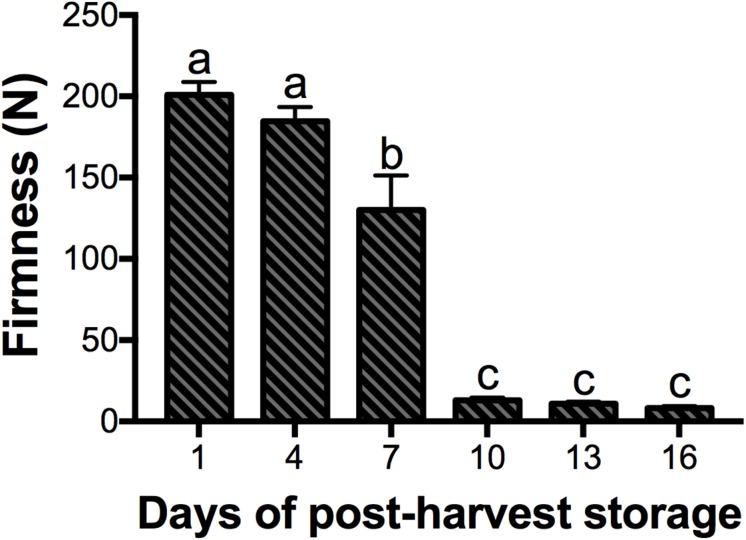
Mango fruit firmness. Data shown represent the average of five biological replicates (*n* = 5). Error bar indicates SE of the means and different letters indicate significant differences. Statistical significance between the control and treatment groups was determined by one-way ANOVA with the Tukey-Kramer test (*P* < 0.05).

Among other changes that occur during ripening of mango fruit, the texture is essential in terms of its relationship with consumer preference, storage, transportation, shelf life and resistance to pathogens (Li et al., 2010). The firmness of the mango cv. Kent fruit is shown in Figure 2. Fruits from days 1 and 4 of post-harvest storage were similar in their firmness. However, at day 7, the firmness of the fruits decreased significantly by 30%, and at day 10, the firmness of fruits was drastically reduced by 90%, remaining constant until the end of ripening (*P* < 0.05). Mango cv. Kent is highly appreciated in the market; however, it has a very fast softening and a short shelf life (Jiménez et al., 2004). Similar results to ours have been reported in other mango varieties such as Harumanis, Kensington Pride and Ataulfo, where reductions of 50, 80, and 75% in mesocarp firmness were observed between 4.5, 6, and 6 days of post-harvest storage, respectively (Ali et al., 2004; Muy Rangel et al., 2009; Razzaq et al., 2016). These textural changes are the result of alterations in the structure and composition of the cell wall, mediated by enzymes related to softening (Brummell, 2006). From the plant cell wall components, the pectins are the most structurally complex and they are essential for cell-to-cell adhesion (Wang et al., 2018). One of the most important enzymes that hydrolyze pectin is PG, which has been extensively studied in a large number of fruits, particularly in tomato (Giovannoni et al., 1989). That is the importance of identifying these enzymes at the molecular level, and in the future, the use of this information might allow quality improvements.

### Transcriptome-Wide and Genome-Wide Identification of PG Genes of Mango Fruit

A total of seventeen *PG* cDNAs were found in the mango mesocarp cv. Kent transcriptome (Dautt-Castro et al., 2015) and they were mapped to the mango cv. Tommy Atkins genome (Dr. DK, personal communication). A total of 48 encoded PGs were found in the mango genome and named after their *Arabidopsis* counterparts (González-Carranza et al., 2007). Those PGs are presented in Table 1, where some characteristics are shown for each identified PG like name, genome identification, GenBank accession number, exon number, protein length and theoretical molecular weight. As summarized in Table 1, the lengths of the mango PGs ranged from 83 aa (MiPG66) to 1916 aa (MiPG69-2) with molecular weights of 8.77 kDa to 210.08 kDa, respectively. PG isoelectric points ranged from 4.77 (MiPG6-1) to 9.27 (MiPG59). Fourty three PGs presented a Cellular Component of “cell wall” and “extracellular region”; meanwhile, five of them were “integral to the membrane.” About the Biological Process, 45 PGs presented “carbohydrate metabolic process,” five also showed “fruit ripening,” one PG showed cell wall organization and one was annotated as “Microsporogenesis/pollen exine formation.” For molecular function, 47 *PG* genes were annotated with “PG activity” (see Supplementary Table S2A). Also, the COG annotation showed that 41 PGs are endo-PGs (COG5434) and seven are exo-PGs (Supplementary Table S2). Interestingly, endo-PGs, whose activity is to endo-degrade homogalacturonans, the primary type of pectins, have been more associated to fruit ripening and softening compared to exo-PGs (Wakasa et al., 2006; Gu et al., 2016; Qian et al., 2016; Wang et al., 2018). On the other hand, 32 of 48 MiPGs (66%) were predicted to have a signal peptide with lengths ranging from 17 to 39 residues (Supplementary Table S2B). Moreover, those MiPGs with a signal peptide were located in the secretory pathway. In tomato, 38 of 54 PGs presented a signal peptide with lengths between 17 to 31 residues, similar to our results (Ke et al., 2018). These signal peptides present in PG amino acid sequences support the fact that they are guided to the cell wall to carry out their hydrolytic function.

**TABLE 1:** Family of polygalacturonases in mango (*Mangifera indica* L.).

***MiPG***	**Genome ID**	**GenBank**	**Exons**	**Localization Chr**	**aa**	**kDa**
***40-1***	Manin00g002060.1	MK936539	16	Chr 0:2,034,254 – 2,069,122	1420	153.28
***40-2***	Manin00g002070.1	MK936540	3	Chr 0:2,075,240 – 2,076,583	388	41.9
***22-2***	Manin00g014940.1	MK936541	5	Chr 0:40,127,372 – 40,129,482	361	38.77
***22-3***	Manin00g014950.1	MK936542	9	Chr 0:40,149,927 – 40,158,394	697	74
***22-7***	Manin02g003450.1	MK936543	4	Chr 2:8,085,713 – 8,087,455	388	41.01
***22-8***	Manin02g003460.1	MK936544	8	Chr 2:8,121,984 – 8,135,247	717	75.52
***22-6***	Manin02g003470.1	MK936545	4	Chr 2:8,164,464 – 8,165,561	222	23.35
***22-5***	Manin02g006220.1	MK936546	4	Chr 2:12,050,377 – 12,052,051	395	41.51
***44-2***	Manin02g010300.1	MK936547	6	Chr 2:16,207,991 – 16,209,888	430	46.24
***46-1***	Manin02g010380.1	MK936548	5	Chr 2:16,272,348 – 16,276,078	455	49.49
***6-1***	Manin02g011060.1	MK936549	6	Chr 2:16,947,539 – 16,949,777	474	51.36
***71-1***	Manin03g008820.1	MK936550	3	Chr 3:14,644,395 – 14,646,459	506	55.11
***49***	Manin04g001200.1	MK936551	5	Chr 4:840,755 – 845,219	444	48.29
***56-2***	Manin04g001870.1	MK936552	6	Chr 4:1,283,689 – 1,286,480	467	51.42
***69-1***	Manin04g008520.1	MK936553	9	Chr 4:6,449,559 – 6,452,110	401	43.57
***69-2***	Manin04g008530.1	MK936554	51	Chr 4:6,453,359 – 6,506,648	1916	210.08
***6-2***	Manin04g015430.1	MK936555	6	Chr 4:15,164,191 – 15,166,702	481	52.04
***46-4***	Manin04g016040.2	MK936556	2	Chr 4:17,641,143 – 17,641,804	126	13.92
***46-2***	Manin04g016050.1	MK936557	5	Chr 4:17,661,281 – 17,664,562	455	49.58
***44-3***	Manin04g016150.1	MK936558	10	Chr 4:17,785,523 – 17,798,068	812	87.45
***58***	Manin05g004560.1	MK936559	5	Chr 5:9,153,656 – 9,159,588	495	55.28
***66***	Manin05g011290.1	MK936560	3	Chr 5:14,468,486 – 14,468,882	83	8.77
***31***	Manin05g011300.1	MK936561	3	Chr 5:14,469,030 – 14,469,864	146	15.58
***52***	Manin06g006140.1	MK936562	5	Chr 6:8,842,198 – 8,853,462	490	53.59
***59***	Manin09g006660.1	MK936563	6	Chr 9:12,050,364 – 12,052,270	265	30.13
***11-1***	Manin09g009540.1	MK936564	6	Chr 9:14,987,782 – 14,989,946	421	45.97
***16***	Manin09g015600.1	MK936565	9	Chr 9:20,563,521 – 20,567,215	439	47.67
***51-1***	Manin11g003610.1	MK936566	7	Chr 11:2,576,944 – 2,581,060	449	50.07
***46-3***	Manin11g004580.1	MK936567	8	Chr 11:3,180,214 – 3,183,340	508	55.04
***42***	Manin11g009310.1	MK936568	12	Chr 11:6,507,441 – 6,517,554	1195	126.7
***10***	Manin11g013560.1	MK936569	14	Chr 11:9,949,786 – 9,957,811	630	68.86
***22-4***	Manin12g011650.1	MK936570	4	Chr 12:14,098,917 – 14,100320	369	39.51
***23-2***	Manin12g011660.1	MK936571	4	Chr 12:14,119,764 – 14,121,825	381	40.32
***23-3***	Manin12g011670.1	MK936572	4	Chr 12:14,130,968 – 14,132,912	396	42.04
***23-1***	Manin12g011680.1	MK936573	2	Chr 12:14,164,835 – 14,165,661	211	21.8
***22-1***	Manin12g011690.1	MK936574	13	Chr 12:14,198,558 – 14,220,187	983	106.04
***21-1***	Manin12g011700.1	MK936575	3	Chr 12:14,234,467 – 4,236,214	320	34.69
***21-2***	Manin12g011710.1	MK936576	4	Chr 12:14,253,498 – 14,255,464	361	38.43
***11-2***	Manin16g003570.1	MK936577	7	Chr 16:9,024,035 – 9,026,214	448	49.13
***71-3***	Manin16g003910.1	MK936578	2	Chr 16:9,392,985 – 9,395,675	628	69.05
***51-2***	Manin16g013070.1	MK936579	6	Chr 16:18,234,739 – 18,238,211	466	51.3
***56-1***	Manin16g014550.1	MK936580	6	Chr 16:19,497,634-19,499,847	481	52.66
***56-3***	Manin17g000810.1	MK936581	6	Chr 17:687,147 – 690,549	488	53.87
***17***	Manin18g005790.1	MK936582	9	Chr 18:4,566,836 – 4,570,385	463	50.55
***71-2***	Manin18g012040.1	MK936583	3	Chr 18:15,695,841 – 15,704,321	470	51.18
***44-1***	Manin19g015630.1	MK936584	7	Chr 19:21,223,404 – 21,227,980	733	79.91
***14***	Manin20g006090.1	MK936585	9	Chr 20:11,233,232 – 11,235,842	457	49.8
***53***	Manin20g008040.1	MK936586	9	Chr 20:2,034,254 – 2,069,122	751	82.41

The ORFs from the 48 mango PG amino acid sequence were aligned to characterize their primary structure. A total of four conserved domains (motifs I, II, III, and IV) that are proposed as essential for PG hydrolysis activity were reported in PG amino acid sequences for several plants (Torki et al., 2000). These different characteristic motifs were found in 29 *M. indica* PGs in their primary structure, within the deduced amino acid sequences (Supplementary Figures S1B,C). Meanwhile, the motif III was absent in the primary structure of 16 PGs (Supplementary Figure S1A), and three sequences showed no domains. Most of the reported PGs for several species do have those four domains conserved. Nonetheless, one or more domains have been lost in some of these proteins, having the third domain turned into the most scarcely conserved (Chen et al., 2016). Aspartic acids in NTD and DD regions are contained in domains I and II, respectively; their carboxylate groups may represent a component of the catalytic site, in any case. The domain III (CGPGHG) seems to participate in the reaction by a histidine as the catalytic residue, whereas the domain IV, formed by residues positively charged, act in ionic interactions with carboxylate groups present in the substrate (Torki et al., 2000; Chen et al., 2016; Ke et al., 2018).

### Phylogenetic, Gene Structure, and Regulatory Regions Analysis of Mango Polygalacturonases

**FIGURE 3:**
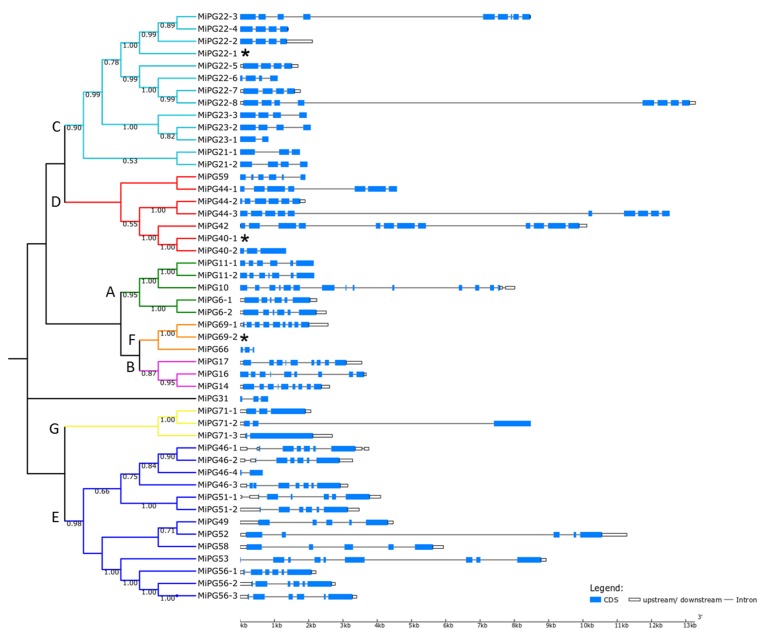
Phylogenetic analysis of PG proteins sequences found in *M. indica* L. Tommy Atkins genome and exon/intron organization of mango PG genes. Left part shows the phylogenetic tree of 48 PG amino acid sequences in *M. indica* L. constructed using MEGA 7 by the NJ method. The number of branches indicates the bootstrap percentage based on 2000 replicates. Values under 50 are not shown in the tree. At the right part, gene structure including exon/intron configuration of each PG is shown and tagged with its respective name. Gene structures for *MiPG22-1*, *MiPG40-1*, and *MiPG69-2* (^∗^) can be seen at Supplementary Figure S3.

A rooted phylogenetic tree composed of 48 mango PG amino acid sequences, previously named according to *A. thaliana* orthologs, was obtained with the Neighbor-Joining tree method and MUSCLE as alignment algorithm (Figure 3 and Supplementary Figure S2). In agreement with Ke et al. (2018), as well as other authors (Yu et al., 2014; Liang et al., 2015), the tree was divided in seven main clades (Clade A to G), except for MiPG31 sequence that appear as an external branch. Interestingly, PG sequences that only presented three functional domains were correctly grouped between clades E, F, and G. It is worth noting that previous studies have shown that clade F members may be associated with flower and probably fruit development (Park et al., 2010; Liang et al., 2015). On the other hand, PG sequences containing all four domains were distributed relatively in the rest of the clades. Remarkably, all PGs classified as exo-PGs (MiPG40-1, MiPG40-2, MiPG44-1, MiPG44-2, MiPG44-3, MiPG42, and MiPG59) were grouped into clade D (see Figure 3), probably because different types of PG have evolved at different times in the history of plants, being the group of exo-PGs one of the most recent and only present in angiosperms (Park et al., 2010).

The gene structures shown in Figure 3 and Supplementary Figure S3 correspond to their full genomic sequences, which show variability in exon/intron numbers within similar or different clades. According to the literature, the number of exons/introns is consistent within the different PGs groups in the tree, resulting in greater exons average number for clades A, B, and F (7.8, 9, and 21, respectively), as it was described before for other species such as *S. lycopersicum*, *Brassica rapa* and *C. sativus* (Yu et al., 2014; Liang et al., 2015; Ke et al., 2018). These results may suggest that across plant evolution PGs sequences have differentiated in each clade, resulting in common and specialized gene structures and biological functions in plants.

Detailed analysis of PG genes in mango cv. Tommy Atkins genome alongside the phylogenetic analysis revealed that all 48 PG genes are present in different chromosomal localizations (Table 1), distributed in all seven clades. Similar to tomato PGs (Ke et al., 2018) all mango PG genes are distributed in most of the chromosomes (Chr 0, 2, 4, 5, 6, 9, 10, 11, 12, 16 y 17). When we compared these findings with the phylogenetic tree (Figure 3), it was found that most of the PG genes that are located in chromosomes 2 and 0 were found in clades C and D. Interestingly, most of the *PGs* located in chromosome 12 were grouped under clade C, and the rest of the *PG* chromosomal localizations were equitably distributed around all clades. Some PG genes were found to be localized side to side, for example, *MiPG22-2* and *MiPG22-3* or *MiPG22-4*, *MiPG23-2*, *MiPG23-3*, *MiPG23-1*, *MiPG22-1*, *MiPG21-1*, and *MiPG21-2* set, all of those are found under the same clade C that contains all four specific domains. On the other hand, *PGs* located in other clades, like *MiPG71-1*, *MiPG72-2*, and *MiPG72-3*, which only contain three of the domains (I, II, and IV) are located in clade G. These results are consistent, for the most part, with gene structure, protein structure, biological function and grouping within the clades.

To further analyze the mango PG sequences, we selected 1.5 Kb upstream of each PG gene and analyzed these regulatory regions. According to MatInspector results, the 48 PGs have in common 20 regulatory regions. Within these, the *Arabidopsis* homeobox protein (P$AHBP), high mobility group factor (P$HMGF), DNA binding with one finger (P$DOFF), CCAAT binding factors (P$CAAT) and soybean embryo factor 4 (P$SEF4) were the most abundant with more than 800 matches (Supplementary Table S3A). Also, the ARID/BRIGHT DNA-binding domain-containing transcription factors (P$ARID), Plant TATA binding protein factor and Yeast TATA binding protein factor (O$YTBP) were the most overrepresented TF in the 48 PGs, compared to *Arabidopsis* genome (Supplementary Table S3B). It is known that some TF are directly related to fruit ripening. In this sense, some MADS-box, like *LeMADS*-*RIN* in tomato are necessary for fruit ripening (Vrebalov et al., 2002). A total of 39 intergenic sequences of mango PGs showed hit with MADS box proteins. Moreover, MADS-box also is implicated in ethylene regulation, which in turn activates several ripening-related genes (Vrebalov et al., 2002). A total of four ethylene insensitive3-like factors (P$EINL), four APETALA2-like transcription factors (P$AP2L) and one ethylene response element factors (P$EREF) were also found within the 48 PGs. Other important TF associated with plant hormone signaling like auxin response element (P$AREF) and auxin response factor 3 (P$ARF3) were also found in 38 and 10 PGs sequences, respectively.

**FIGURE 4:**
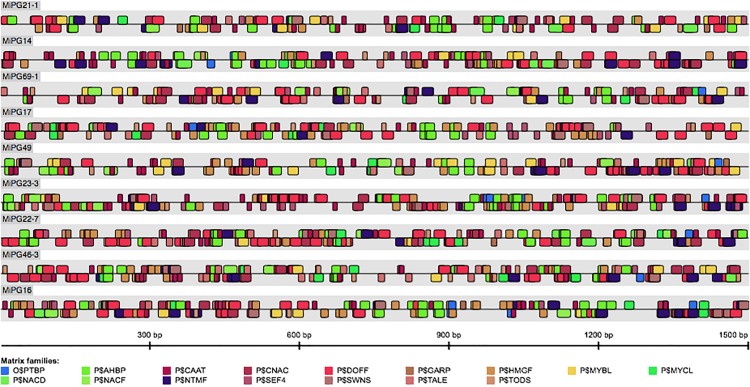
Identification of *cis*-regulatory elements in the 1500 pb promoter region analyzed for the 9 PGs which relative expression was evaluated. The MatInspector program was used, core promoter elements and plant matrix groups, with 0.90 of core and matrix similarity.

Additionally, the 9 PGs which relative expression was evaluated shared 15 *cis*-regulatory elements in the 1500 pb promoter region analyzed (Figure 4 and Supplementary Table S3C). Among these, P$DOFF family was one of the most abundant with 285 matches. Interestingly, in banana fruit (*Musa acuminata*) it has been shown that four Dof transcription factors (MaDof10, 23, 24, and 25) that preferentially bind to the core sequence 5′-(T/A)AAAG-3′ belonging to P$DOFF elements, are ethylene-inducible, and their transcripts are accumulated during ripening. Also, MaDof23 interact with MaERF9 (regulator of fruit ripening), acting as a transcriptional repressor, whereas MaERF9 is a transcriptional activator. This antagonistic relationship, lead to the regulation of ripening-related genes, including *MaPG1* (Yanagisawa, 2002; Feng et al., 2016). On the other hand, the presence of NAC *cis*-regulatory sites may play a main role in PGs expression. In the 9 PGs, several *cis* elements with NAC domain were found (i.e., P$NTMF, P$CNAC, P$NACD, P$NACF, and P$SWNS). In this sense, NAC gene family has been recognized as important transcription factors involved in ripening and softening in fruits like citrus, banana, tomato and peach (de Oliveira et al., 2011; Shan et al., 2012; Zhu et al., 2014; Zhou et al., 2015). Also, in *Fragaria chiloensis* FcNAC1 interact with DNA sequences containing NAC binding elements in the promoter of a pectate lyase *FcPL*, activating its transcription (Carrasco-Orellana et al., 2018). Otherwise, each of 9 mango PGs showed at least 4 elements of P$GARP family in their promoter regions. Evidence point out that a transcription factor of the GARP family acts together with bZIP transcription factor PERIANTHIA to activate the transcription of AGAMOUS, an important member of fruit ripening regulation (Maier et al., 2009). Together, these results suggest that PGs whose gene expression was evaluated, may be regulated by members of these transcription factors that bind to the predicted *cis* elements; moreover, their expression occurs during the advance of the ripening state. However, further studies must be performed in order to prove these asseverations.

### Nine PGs Were Differentially Expressed During Post-harvest Ripening and That Correlated With PG Enzymatic Activity

**FIGURE 5:**
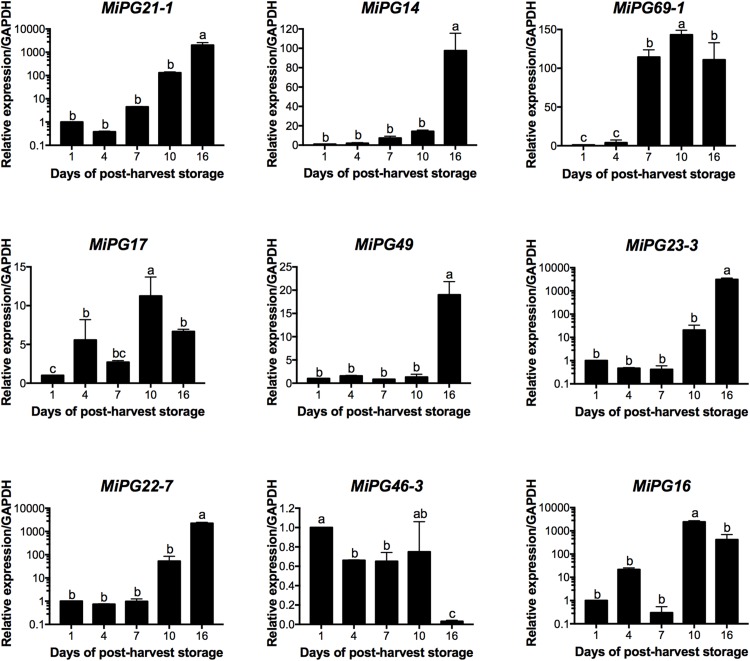
Relative expression of *MiPG21-1*, *MiPG14*, *MiPG69-1*, *MiPG17*, *MiPG49*, *MiPG23-3*, *MiPG22-7*, *MiPG46-3*, and *MiPG16* in mango mesocarp. Data of the nine genes represent the average of three biological replicates (*n* = 3) with three experimental replicates each. Error bars indicate SE of the means and different letters indicate significant differences. Statistical significance of differences between days of post-harvest storage was determined by one-way ANOVA with the Fisher test (*P* < 0.05).

**FIGURE 6:**
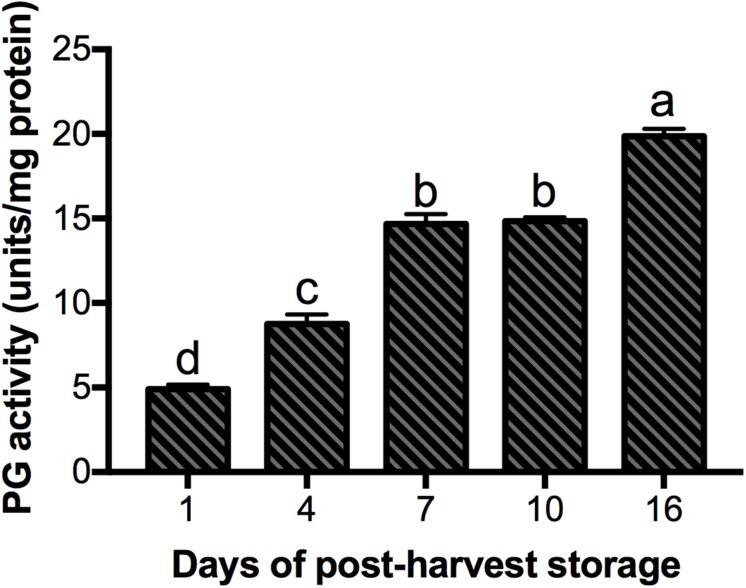
Polygalacturonase (PG) activity in “Kent” mango fruit during storage at 20°C. Error bars indicate SD of the means (*n* = 3). Different letters indicate statistically significant differences between days of post-harvest storage using one-way ANOVA and Tuckey-Kramer test (*P* < 0.05).

According to the transcriptome data (Dautt-Castro et al., 2015), nine *PG* genes were differentially expressed during fruit ripening and selected to validate their expression by qRT-PCR (Figure 5). For this validation, we used two reference genes (*GAPDH* and *ACT 7*) in order to normalize the data. In both analyses the expression patters for eight PGs evaluated followed the same trends with similar expression levels, which support our results (Figure 5 and Supplementary Figure S4). In this regard, five *PG* genes (*MiPG21-1, MiPG14, MiPG49, MiPG23-3, MiPG22-7*) presented their maximum level of expression at 16 days of post-harvest storage; meanwhile for *MiPG69-1, MiPG17*, and *MiPG16* maximum levels were at day 10. *MiPG46-3* was down-regulated in mango from day 4, 7, and 16; however, in fruit from day 1 and 10 it remained at similar levels. The transcript with highest relative expression was *MiPG23-3* with 3131-fold at day 16 followed by *MiPG16* with 2426-fold at day 10. These results were very similar to the expression ratio found in transcriptome by RNA-seq (Supplementary Figure S5). PGs from clade C (MiPG21-1, MiPG22-7, and MiPG23-3) are among those that presented the highest relative expression levels in mango from day 16 of post-harvest (2,000-fold). MiPG16 (clade B) also presented very high relative expression levels (3,000-fold). Meanwhile, MiPG69-1 (clade F) presented relative expression levels of about 200-fold in mango from day 7 of post-harvest when firmness was reduced by 30%. Also, MiPG14 (clade B) that is closely related to MiPG69-1 (see Figure 3) was expressed at 100-fold but in fruit from day 16 after post-harvest. MiPG49 (also from clade B) was 20-fold in mango from day 16, and MiPG17 was 12-fold in fruit from day 10 after post-harvest. These results suggest that these PGs could be related to the loss of firmness observed in the mango fruit. The up-regulation of these *PGs* during the advance of mango ripening correlates with the activity of PG (Figure 6), where the activity increased with the advance of ripening. In mango from day 1 and 4 of post-harvest, PG activity was 5 and 9 units/mg protein; while for mango of days 7 and 10 it was 15 units/mg protein and in mango from day 16 of post-harvest the maximum PG activity was reached (19.8 units/mg protein).

The expression of *PG* genes has been extensively studied in other fruits. Furthermore, it has been observed that *PG* genes related to fruit ripening, often are induced by the endogenous production of ethylene and the application of exogenous ethylene. As found in the expression of almost all evaluated mango *PGs* (except for *MiPG46*-3), their expressions levels increased with the advance of ripening. The differential expression of *PG* cDNAs has also been observed in fruits such as bananas, where four PGs associated with maturation (*MaPG1, MaPG2, MaPG3*, and *MaPG4*) were evaluated in the fruit peel. Maximum expression levels were observed at days 4, 2, 7, and 7 of post-harvest, respectively; featuring the highest levels of gene expression in *MaPG4* (values of about 2300-fold, 2^–ΔΔCt^) and the lowest in *MaPG3* (values of approximately 1.3-fold, 2^–ΔΔCt^) (Mbéguié-A-Mbéguié et al., 2009). The expression of the apricot ortholog gene of peach PRF5 named *PaPG* was evaluated in three different varieties (Goldrich, Currot, and Canino) and its expression correlated with fruit softening and ethylene release; moreover, *PaPG* responded to exogenous ethylene (Leida et al., 2011). The results obtained for mango PGs suggest that these enzymes could be ethylene-dependent, which correlates with the ethylene-response elements found in many PG promoter regions (Supplementary Table S3) and suggest that them could be associated with ripening and responsible in part for the softening on mango fruits.

As described above, the maximum loss of firmness of mango fruit was registered at day 10 of post-harvest storage. Different studies have shown that the silencing of independent cell wall-associated genes including PGs (Smith et al., 1988), pectin methylesterases (Phan et al., 2007), pectate lyases (Uluisik et al., 2016) and expansins (Brummell et al., 1999) have resulted in a range of effects of softening, and in some cases with zones still rich in de-esterified pectins (Yang et al., 2017). This evidence suggests that the loss of firmness is dependent on the action of different families of cell wall-degrading enzymes that act in distinct ripening stages. In this sense, we could infer that the evaluated mango PGs are related to loss of firmness in the fruit. Therefore, the relationship of the gene expression data found and analyzed in this study as well as the physiological parameters of mango cv. “Kent,” during their maturation process, show evidence of the role that *PG* genes associated with this process, specifically in fruit softening. Thereby also demonstrating the importance of these genes in the ripening process of fruits.

### PG Structural Models

**FIGURE 7:**
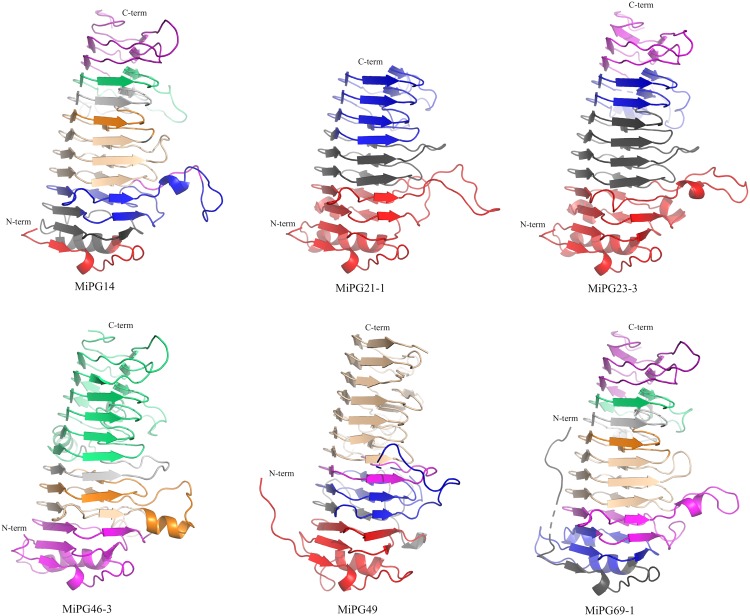
Structural models for selected mango PGs. The structures are colored according to the exon color used in Supplementary Figure S6. MiPG14 (clade B), MiPG69-1 (clade F), MiPG21-1 and MiPG23-3 (clade C), MiPG46-3, and MiPG49 (clade E) were modeled using rhamnogalacturonase A from *Aspergillus aculeatus* (PDB 1RMG) as a template. Figures were built using PyMol.

Mango PGs are structurally conserved (Figure 7) and have a single-stranded right-handed beta helix structure, also known as a pectin lyase-like CATH superfamily^5^ (Dawson et al., 2017; Lewis et al., 2018). This superfamily is mostly found in bacteria, plant and fungus, and scarcely on invertebrates and environmental samples. According to biochemical functions, this fold encompasses 63% of pectin esterases (E.C. 3.1.1.11), followed by pectate lyases (16%, E.C. 4.2.2.2). All enzymes having the parallel beta-helical fold can recognize and hydrolyze large polysaccharides (Fujimoto, 2013).

The structures for the six PGs are very similar; for example, MiPG14 (clade B) and MiPG69-1 (clade F) have 9 exons in their gene structures, and they share a similar exon distribution and structural arrangement (Supplementary Figure S6). Interestingly, this result supports the hypothesis that exons code for functional structural units in proteins (Traut, 1988), and structural models may help to understand the evolutionary history of PGs in mango. MiPG21-1 and MiPG23-3 are from clade C, with three and four exons, respectively; their structural models are similar; although, MiPG23-3 is 76 residues longer and the fourth exon leads to a larger structure. MiPG46-3 and MiPG49 pertain to clade E, and have 8 and 5 exons, respectively; their models present differences in exon distribution, although overall the structural models are very similar. Until now, there is no crystallographic structure for a plant PG; all solved structures are mostly from fungi and one from bacteria. One important aspect to further explore the structural study of these proteins is the accommodation of the polysaccharide into each active site. Another area of interest would be the biochemical characterization of the PGs in terms of their kinetic properties.

Our results add knowledge about this multigenic family of enzymes involved in structural changes in the cell wall and the concomitant softening of the fruit. Beyond physiological aspects, molecular information about gene expression and evolutive relationships of mango PGs, this work add some perspectives about the study of PG in climacteric fruits, that could be used to improve also shelf-life of other plants species.

## Data Availability

The datasets generated for this study can be accessed from GenBank MK936539-MK936586. The datasets analyzed for this study can be found deposited in the NCBI (Accesion PRJNA258477) (https://www.ncbi.nlm.nih.gov/bioproject/PRJNA258477).

## Author Contributions

MI-O and MD-C conceived and designed the experiments. MD-C performed the experiments. MD-C, AL-V, AO-L, MI-O, CC-V, DK, AS-S, and AS-B analyzed the data. MI-O, AO-L, GG-A, JC-F, MM-T, and AS-B contributed to the reagents, materials, and analysis tools. MD-C, MI-O, CC-V, AL-V, AS-S, and DK wrote the manuscript. All authors reviewed the manuscript.

## Conflict of Interest Statement

The authors declare that the research was conducted in the absence of any commercial or financial relationships that could be construed as a potential conflict of interest.

## Footnotes

1 http://web.expasy.org/compute_pi/

2 https://embnet.vital-it.ch/software/BOX_form.html

3 http://www.cbs.dtu.dk/services/SignalP/

4 http://gsds.cbi.pku.edu.cn/

5 http://www.cathdb.info/version/latest/superfamily/2.160.20.10

## Supplementary Material

FIGURE S1 Multiple sequence alignment analysis of the polygalacturonases (PGs) from mango (*Mangifera indica* L.). Partial sequences where the characteristic domains are located are shown, (1A) Shows PGs that contain motifs I, II and IV; (1B) and (1C) Show PGs that have motifs I, II, II and IV. Red boxes show the four functional motifs. Identical amino acids are shown in black, and gray shading represents conserved amino acid residues.

FIGURE S2 Phylogenetic analysis of *Mangifera indica* L. and *Arabidopsis thaliana* polygalacturonases. The evolutionary history was inferred using the Neighbor-Joining method, while distances were computed using the Poisson correction method. The analysis involved 117 amino acid sequences, of which 48 sequences belong to mango (*MiPGs*). Taxa clustered together in the bootstrap test (2000 replicates). Evolutionary analyses were conducted in MEGA X.

FIGURE S3 Gene structure of the longest mango PG genes including exon/intron configuration. *MiPG40-1* contains 16 exons, *MiPG69-2* contains 51 exons and *MiPG22-1* has 13 exons. *MiPG40-1* encodes an enzyme of 1420 aa, *MiPG69-2* and enzyme of 1916 aa and *MiPG22-1* an enzyme of 983 aa long. The structure of the introns and exons of the *MiPG* genes were obtained using GSDS 2.0 (http://gsds.cbi.pku.edu.cn/).

FIGURE S4 Relative expression of *MiPG21-1, MiPG14, MiPG69-1, MiPG17, MiPG49, MiPG23-3, MiPG22-7, MiPG46-3*, and *MiPG16* in mango mesocarp, using *ACT 7* as reference gene. Data of the nine genes represent the average of three biological replicates (*n* = 3) with three experimental replicates each. Error bars indicate SE of the means and different letters indicate significant differences. Statistical significance of differences between days of postharvest storage was determined by one-way ANOVA with the Fisher test (*P* < 0.05).

FIGURE S5 Expression ratio (Log_2_) for the 9 polygalacturonases was obtained by RNA-seq and qPCR. *GAPDH* was the constitutive gene used for normalization of qPCR data. Bars represent the standard error (*n* = 3).

FIGURE S6 Sequence alignment of polygalacturonases that were modeled to obtain theoretical models, the exons were colored with different colors. MiPG21-1 and MiPG23-3 are from clade C, MiPG14 from clade B, MiPG69-1 from clade F; while MiPG46-3 and MiPG49 are from clade E.

TABLE S1 Primer sequences used for qRT-PCR.

TABLE S2 PG superfamilies identified in mango polygalacturonases. **(A)** Genome ID, sequence name, biological process, cellular component, molecular function, classification, enzyme kind (endo/exo), isoelectric point and GenBank accession. **(B)** SignalP analysis of polygalacturonases from mango fruit.

TABLE S3 Analysis of regulatory regions in 5′UTR polygalacturonases genes. **(A)** Common regulatory regions identified in 48 PGs. **(B)** Overrepresented TF families. **(C)** Common regulatory regions identified in 9 PGs which expression was evaluated.
