# Supplementary material for: Genome-Wide Identification of Mango (Mangifera indica L.) Polygalacturonases: Expression Analysis of Family Members and Total Enzyme Activity During Fruit Ripening
Source: Front Plant Sci. 2019 Jul 30;10:969. doi: 10.3389/fpls.2019.00969 (PMC6682704; doi:10.3389/fpls.2019.00969)
Supplement: FIGURE S1 — Multiple sequence alignment analysis of the polygalacturonases (PGs) from mango (Mangifera indica L.). Partial sequences where the characteristic domains are located are shown, (1A) Shows PGs that contain motifs I, II and IV; (1B) and (1C) Show PGs that have motifs I, II, II and IV. Red boxes show the four functional motifs. Identical amino acids are shown in black, and gray shading represents conserved amino acid residues. [file Image_1.pdf]

Supplementary Material

1 Supplementary Figures

A)

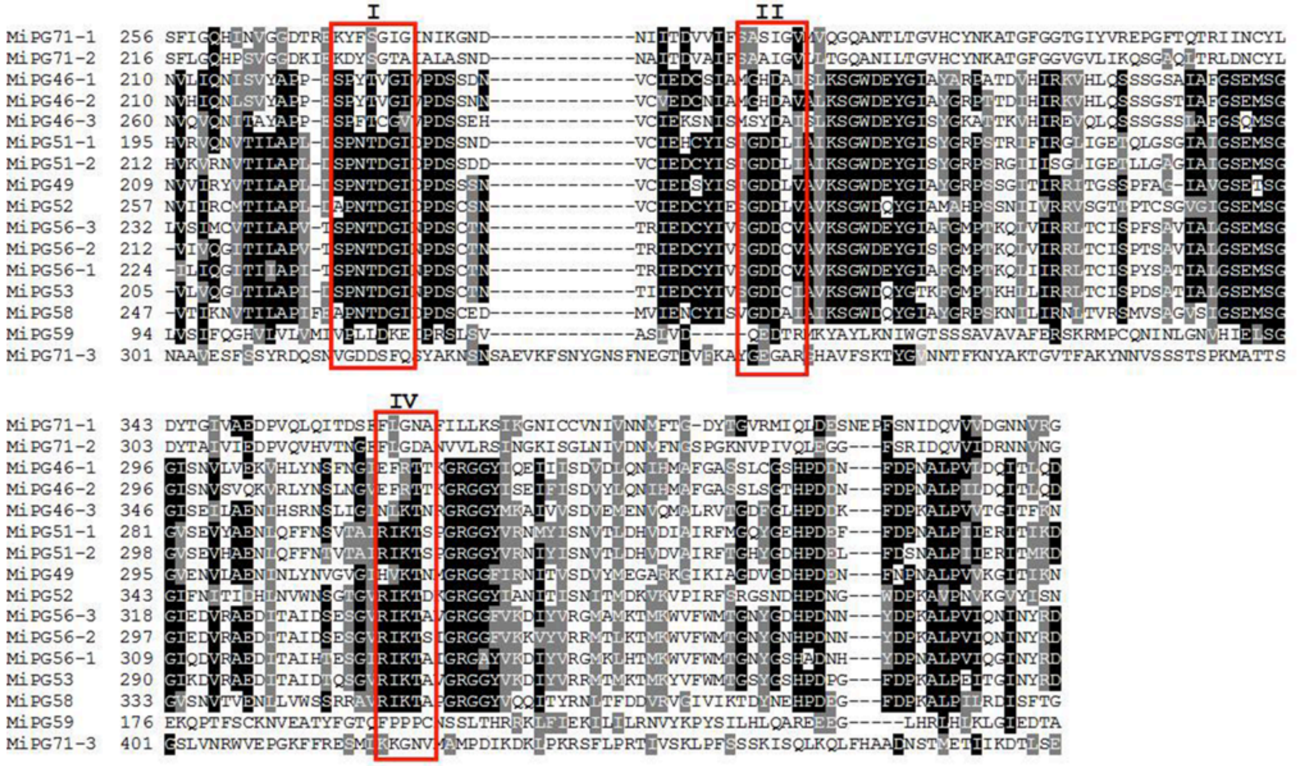

B)

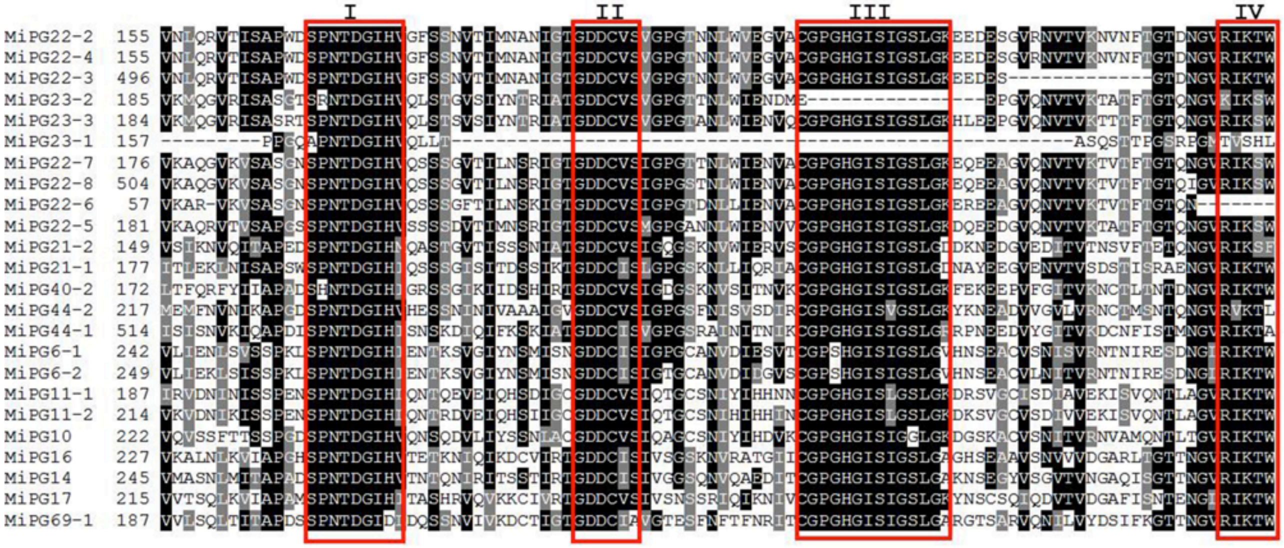

C)

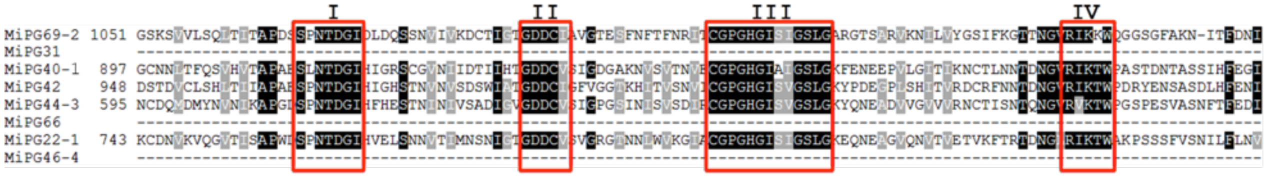

Supplementary Figure S1. Multiple sequence alignment analysis of the polygalacturonases (PGs) from mango (*Mangifera indica* L.). Partial sequences where the characteristic domains are located are shown, A) Shows PGs that contain motifs I, II and IV; B) and C) Show PGs that have motifs I, II, II and IV. Red boxes show the four functional motifs. Identical amino acids are shown in black, and gray shading represents conserved amino acid residues.
