# Supplementary material for: Genome-Wide Identification of Mango (Mangifera indica L.) Polygalacturonases: Expression Analysis of Family Members and Total Enzyme Activity During Fruit Ripening
Source: Front Plant Sci. 2019 Jul 30;10:969. doi: 10.3389/fpls.2019.00969 (PMC6682704; doi:10.3389/fpls.2019.00969)
Supplement: FIGURE S2 — Phylogenetic analysis of Mangifera indica L. and Arabidopsis thaliana polygalacturonases. The evolutionary history was inferred using the Neighbor-Joining method, while distances were computed using the Poisson correction method. The analysis involved 117 amino acid sequences, of which 48 sequences belong to mango (MiPGs). Taxa clustered together in the bootstrap test (2000 replicates). Evolutionary analyses were conducted in MEGA X. [file Image_2.pdf]

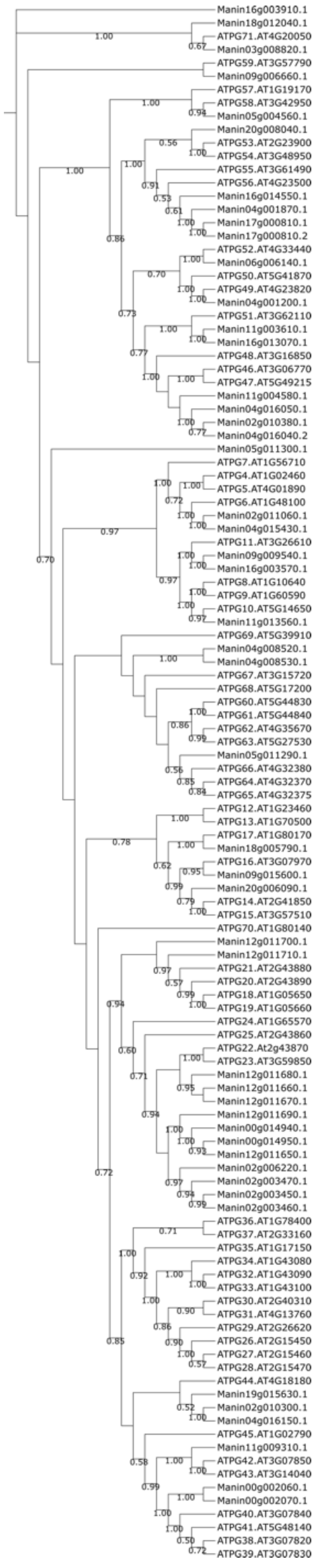
Manin16g003910.1
Manin18g012040.1
ATPG71.AT4G20050
Manin03g008820.1
ATPG59.AT3G57790
Manin09g006660.1
ATPG57.AT1G19170
ATPG58.AT3G42950
Manin05g004560.1
Manin20g008040.1
ATPG53.AT2G23900
ATPG54.AT3G48950
ATPG55.AT3G61490
ATPG56.AT4G23500
Manin16g014550.1
Manin04g001870.1
Manin17g000810.1
Manin17g000810.2
ATPG52.AT4G33440
Manin06g006140.1
ATPG50.AT5G41870
ATPG49.AT4G23820
Manin04g001200.1
ATPG51.AT3G62110
Manin11g003610.1
Manin16g013070.1
ATPG48.AT3G16850
ATPG46.AT3G06770
ATPG47.AT5G49215
Manin11g004580.1
Manin04g016050.1
Manin02g010380.1
Manin04g016040.2
Manin05g011300.1
ATPG7.AT1G56710
ATPG4.AT1G02460
ATPG5.AT4G01890
ATPG6.AT1G48100
Manin02g011060.1
Manin04g015430.1
ATPG11.AT3G26610
Manin09g009540.1
Manin16g003570.1
ATPG8.AT1G10640
ATPG9.AT1G60590
ATPG10.AT5G14650
Manin11g013560.1
ATPG69.AT5G39910
Manin04g008520.1
Manin04g008530.1
ATPG67.AT3G15720
ATPG68.AT5G17200
ATPG60.AT5G44830
ATPG61.AT5G44840
ATPG62.AT4G35670
ATPG63.AT5G27530
Manin05g011290.1
ATPG66.AT4G32380
ATPG64.AT4G32370
ATPG65.AT4G32375
ATPG12.AT1G23460
ATPG13.AT1G70500
ATPG17.AT1G80170
Manin18g005790.1
ATPG16.AT3G07970
Manin09g015600.1
Manin20g006090.1
ATPG14.AT2G41850
ATPG15.AT3G57510
ATPG70.AT1G80140
Manin12g011700.1
Manin12g011710.1
ATPG21.AT2G43880
ATPG20.AT2G43890
ATPG18.AT1G05650
ATPG19.AT1G05660
ATPG24.AT1G65570
ATPG25.AT2G43860
ATPG22.At2g43870
ATPG23.AT3G59850
Manin12g011680.1
Manin12g011660.1
Manin12g011670.1
Manin12g011690.1
Manin00g014940.1
Manin00g014950.1
Manin12g011650.1
Manin02g006220.1
Manin02g003470.1
Manin02g003450.1
Manin02g003460.1
ATPG36.AT1G78400
ATPG37.AT2G33160
ATPG35.AT1G17150
ATPG34.AT1G43080
ATPG32.AT1G43090
ATPG33.AT1G43100
ATPG30.AT2G40310
ATPG31.AT4G13760
ATPG29.AT2G26620
ATPG26.AT2G15450
ATPG27.AT2G15460
ATPG28.AT2G15470
ATPG44.AT4G18180
Manin19g015630.1
Manin02g010300.1
Manin04g016150.1
ATPG45.AT1G02790
Manin11g009310.1
ATPG42.AT3G07850
ATPG43.AT3G14040
Manin00g002060.1
Manin00g002070.1
ATPG40.AT3G07840
ATPG41.AT5G48140
ATPG38.AT3G07820
ATPG39.AT3G07830

Supplementary Figure S2. Phylogenetic analysis of *Mangifera indica* L. and *Arabidopsis thaliana* polygalacturonases. The evolutionary history was inferred using the Neighbor-Joining method, while distances were computed using the Poisson correction method. The analysis involved 117 amino acid sequences, of which 48 sequences belong to mango (*MiPGs*). Taxa clustered together in the bootstrap test (2000 replicates). Evolutionary analyses were conducted in MEGA X.
