# Supplementary material for: Genome-Wide Identification of Mango (Mangifera indica L.) Polygalacturonases: Expression Analysis of Family Members and Total Enzyme Activity During Fruit Ripening
Source: Front Plant Sci. 2019 Jul 30;10:969. doi: 10.3389/fpls.2019.00969 (PMC6682704; doi:10.3389/fpls.2019.00969)
Supplement: FIGURE S3 — Gene structure of the longest mango PG genes including exon/intron configuration. MiPG40-1 contains 16 exons, MiPG69-2 contains 51 exons and MiPG22-1 has 13 exons. MiPG40-1 encodes an enzyme of 1420 aa, MiPG69-2 and enzyme of 1916 aa and MiPG22-1 an enzyme of 983 aa long. The structure of the introns and exons of the MiPG genes were obtained using GSDS 2.0 (http://gsds.cbi.pku.edu.cn/). [file Image_3.pdf]

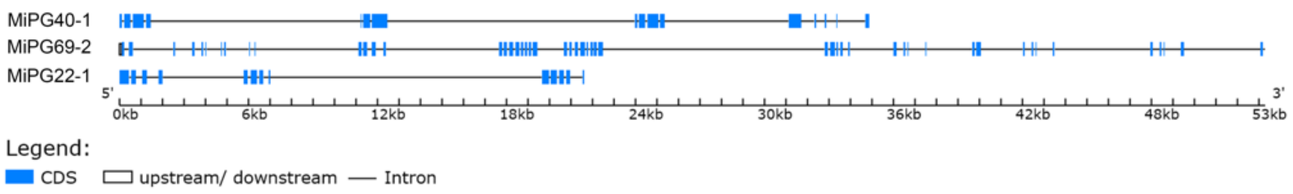

Supplementary Figure S3. Gene structure of the longest mango PG genes including exon/intron configuration. *MiPG40-1* contains 16 exons, *MiPG69-2* contains 51 exons and *MiPG22-1* has 13 exons. *MiPG40-1* encodes an enzyme of 1420 aa, *MiPG69-2* and enzyme of 1916 aa and *MiPG22-1* an enzyme of 983 aa long. The structure of the introns and exons of the *MiPG* genes were obtained using GSDS 2.0 (http://gsds.cbi.pku.edu.cn/).
