# Supplementary material for: Genome-Wide Identification of Mango (Mangifera indica L.) Polygalacturonases: Expression Analysis of Family Members and Total Enzyme Activity During Fruit Ripening
Source: Front Plant Sci. 2019 Jul 30;10:969. doi: 10.3389/fpls.2019.00969 (PMC6682704; doi:10.3389/fpls.2019.00969)
Supplement: FIGURE S4 — Relative expression of MiPG21-1, MiPG14, MiPG69-1, MiPG17, MiPG49, MiPG23-3, MiPG22-7, MiPG46-3, and MiPG16 in mango mesocarp, using ACT 7 as reference gene. Data of the nine genes represent the average of three biological replicates (n = 3) with three experimental replicates each. Error bars indicate SE of the means and different letters indicate significant differences. Statistical significance of differences between days of postharvest storage was determined by one-way ANOVA with the Fisher test (P < 0.05). [file Image_4.pdf]

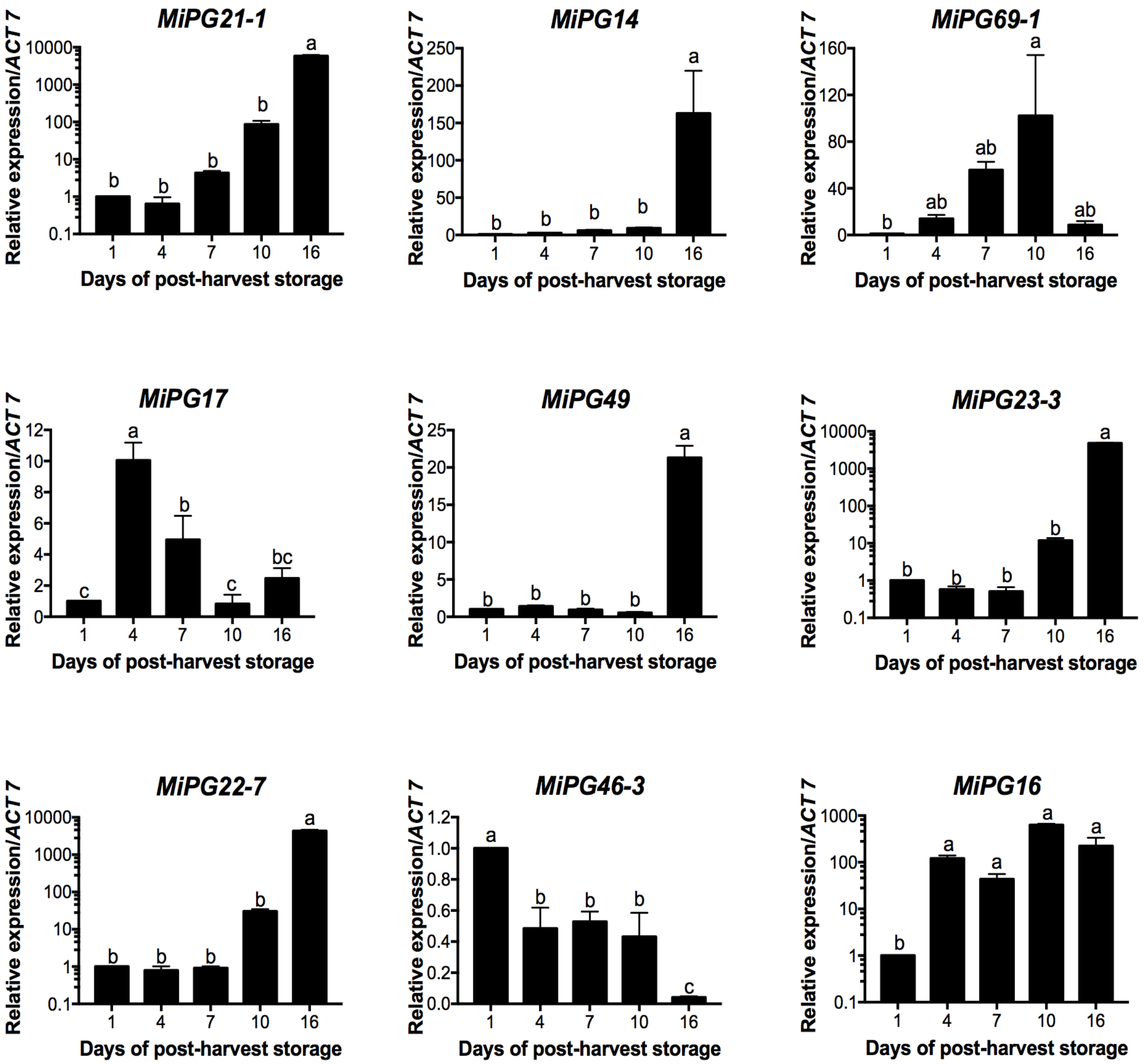

Supplementary Figure 4. Relative expression of *MiPG21-1, MiPG14, MiPG69-1, MiPG17, MiPG49, MiPG23-3, MiPG22-7, MiPG46-3* and *MiPG16* in mango mesocarp, using *ACT 7* as reference gene. Data of the nine genes represent the average of three biological replicates (n=3) with three experimental replicates each. Error bars indicate SE of the means and different letters indicate significant differences. Statistical significance of differences between days of postharvest storage was determined by one-way ANOVA with the Fisher test *($P<0.05$)*.
