# Supplementary material for: Genome-Wide Identification of Mango (Mangifera indica L.) Polygalacturonases: Expression Analysis of Family Members and Total Enzyme Activity During Fruit Ripening
Source: Front Plant Sci. 2019 Jul 30;10:969. doi: 10.3389/fpls.2019.00969 (PMC6682704; doi:10.3389/fpls.2019.00969)
Supplement: FIGURE S5 — Expression ratio (Log2) for the 9 polygalacturonases was obtained by RNA-seq and qPCR. GAPDH was the constitutive gene used for normalization of qPCR data. Bars represent the standard error (n = 3). [file Image_5.pdf]

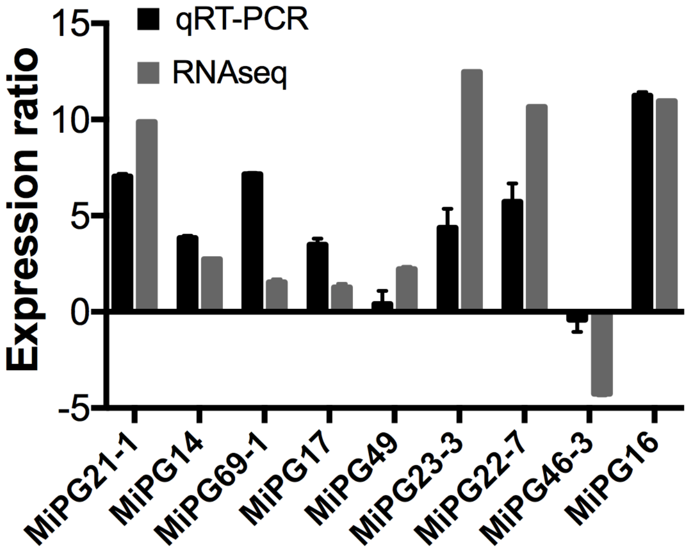

Supplementary Figure 5. Expression ratio (Log_2) for the 9 polygalacturonases was obtained by RNA-seq and qPCR. *GAPDH* was the constitutive gene used for normalization of qPCR data. Bars represent the standard error (n=3).
