# Supplementary material for: Genome-Wide Identification of Mango (Mangifera indica L.) Polygalacturonases: Expression Analysis of Family Members and Total Enzyme Activity During Fruit Ripening
Source: Front Plant Sci. 2019 Jul 30;10:969. doi: 10.3389/fpls.2019.00969 (PMC6682704; doi:10.3389/fpls.2019.00969)
Supplement: FIGURE S6 — Sequence alignment of polygalacturonases that were modeled to obtain theoretical models, the exons were colored with different colors. MiPG21-1 and MiPG23-3 are from clade C, MiPG14 from clade B, MiPG69-1 from clade F; while MiPG46-3 and MiPG49 are from clade E. [file Image_6.pdf]

```
MiPG21-1    1 ------------MSKLSVSCKFFIISFLTLFQLTVEAYN---------------------
MiPG23-3    1 ------MENKTSYNLLLLLASLNLAFLFSSWSASASSFN---------------------
MiPG14      1 ---MALLHPYASFTFSIIFIPFILSYFCLSVKDTPYDYYHQEAYGYGHDSLAYPSHLTAI
MiPG69-1    1 ------MKLKIFTTFMVLSMAHCGSSLQLSLKPRAFEYS---------------------
MiPG49      1 ---MARVFLLFSLILLSYVSSFSLASVITCSGIVPLRYR---------------------
MiPG46-3   61 SVSYLPVVLLLVLTLSGAAEQNVKESDGHCKYARSLEPR---------------------

MiPG21-1   28 ----------------------------------VISFGAKPDGQTDSTQAFANAWESAC
MiPG23-3   34 ----------------------------------VVSFGAKADGKSDSTKAFLDAWAQAC
MiPG14     58 EDAEFEEYLIKLMRKEILGSNVVGLFSTAKNTVIVDDFGAKGDGSTDDTEAFKKAWQEAC
MiPG69-1   34 --------------------------------VNVMNMGAVGDGTTDDSQAFLKAWAAVC
MiPG49     37 -----------------------------NDTISITDFGGVGDGKTLNTKAFREAIYRIE
MiPG46-3  100 -----------------------------PHSVSLTEFGAVGDGKTLNTVAFQNAIFYLK

MiPG21-1   54 KSTRPATINVPKG--RFLIKPIYFNGTCN-NEIIFQINGTIVAPASYSSLG-DSGFWIIF
MiPG23-3   60 GSATAATVYVPKG--RFLLRNSVFAGQCKSTKITVRIDGTLVAPSDYNVIG-NSKYWLLF
MiPG14    118 SQG---AVLVVPGDKTYYLKPIRFSGPCKPN-LMVEIYGTLEASDSQSDYRKDRKHWLLF
MiPG69-1   62 GAQKSEPLVVIPGGKTFLLYPVTFRGPCSSSSVNIQIWGNIVAPEYSAWKGKDVQTWLLF
MiPG49     68 RLRRRGGTLLYVPSGVYLTESFNLTSHMTLYLARGAVIKATQDTDNWPLIAPLPSYGRGR
MiPG46-3  131 SFADKGGAQLYVPPGKWLTGSFNLTSHLTLFLERGAVILGSKDYSHWEVVEPLPSYGRGI

MiPG21-1  110 YRVSG---LIVRG------------GTFDAKGAAYWDCR---KSGKSCPSLPRSVTFMES
MiPG23-3  117 QQVNT---VTING------------GILDGQGTGLWACK---NSGKSCPGGATSISFMNS
MiPG14    174 DSVEN---LLVEGG-----------GTINGNGRIWWQNSCKVNKSLPCKDAPTALTFYRS
MiPG69-1  122 DSINW---LKIDGN-----------GQIDGRGESWWKN------AYSPYNRPSALTFNNC
MiPG49    128 ELPGGRYMSFIHGDGLHDVVITGENGTIDGQGAIWWNMWR---QRTLPFTRPNLIEFINS
MiPG46-3  191 DLPGGRYRALINGN------------NLIDVGSVWWELFN---SGDLKYSRPHLVEFTGS

MiPG21-1  152 SNVVVSRLKSLNSRYFHIAIDGCENITLEKLNISAPSWSPNTDGIHIQSSSGISITDSSI
MiPG23-3  159 NNIVINGLTSQNSQLYHIVFNGCNNVKMQGVRISASRTSPNTDGIHVQLSTSVSIYNTRI
MiPG14    220 KNLVVRNLNIQDSQQIHVSFQKSTNVMASNLMITAPADSPNTDGIHVTNTQNIRITSSTI
MiPG69-1  162 NNLHVNGLHHVNSQRNHITVTGSKSVVLSQLTITAPDSSPNTDGIDLDQSSNVIVKDCTI
MiPG49    185 RSIIISNVIFQNSPFWNIHPVYCSNVVIRYVTILAPLDSPNTDGIDPDSSSNVCIEDSYI
MiPG46-3  236 NDVTISNLTFLNSPAWNIHPVYCSNVQVQNITAYAPPESPFTCGVVPDSSEHVCIEKSNI

MiPG21-1  212 KTGDDCISLGPGSKNLLIQRIACGPGHGISIGSLGDNAY---------EEGVENVTVSDS
MiPG23-3  219 ATGDDCVSVGPGTANLWIENVQCGPGHGISIGSLGKHLE---------EPGVQNVTVKTT
MiPG14    280 RTGDDCISIVGGSQNVQAEDITCGPGHGISIGSLGAKNS---------EGYVSGVTVNGA
MiPG69-1  222 GTGDDCIAVGTESFNFTFNRITCGPGHGISIGSLGARGT---------SARVQNILVYDS
MiPG49    245 STGDDLVAVKSGWDEYGIAYGRPSSGITIRRITG-SSPFAGIAVGSETSGGVENVLAENI
MiPG46-3  296 SMSYDAISLKSGWDEYGISYGKATTKVHIREVQLQSSSGSSLAFGSQMSGGISEILAENI

MiPG21-1  263 TISRAENGVRIKTWARSSNGFAKNINFQNIIMKNAYNPILIDQYYCPGHKGCPNQVIN--
MiPG23-3  270 TFTGTQNGVRIKSWGRPSNGFARNILFQHAVMSNVQNPIIIDQNYCPDNINCPGRVSGVK
MiPG14    331 QISGTTNGVRIKTWQGGS-GTASNIKFQNIYMFNVSNPIIIDQNYCDQEEPCKEQSSAVQ
MiPG69-1  273 IFKGTTNGVRIKTWQGGS-GSAKNITFDKIRLESVQNPIIIDQYYCPHRN-CQNQTSAVR
MiPG49    304 NLYNVGVGIHVKTNMGRG-GFIRNITVSDVYMEGARKGIKIAGDVGDHPDENFNPNALPV
MiPG46-3  356 HSRNSLIGINLKTNRGRG-GYMKAIVVSDVEMENVQMALRVTGDFGLHPDDKFDPKALPV
```

Supplementary Figure S6. Sequence alignment of polygalacturonases that were modeled to obtain theoretical models, the exons were colored with different colors. MiPG21-1 and MiPG23-3 are from clade C, MiPG14 from clade B, MiPG69-1 from clade F; while MiPG46-3 and MiPG49 are from clade E.
