# Supplementary material for: Genome-Wide Identification of Mango (Mangifera indica L.) Polygalacturonases: Expression Analysis of Family Members and Total Enzyme Activity During Fruit Ripening
Source: Front Plant Sci. 2019 Jul 30;10:969. doi: 10.3389/fpls.2019.00969 (PMC6682704; doi:10.3389/fpls.2019.00969)
Supplement: TABLE S1 — Primer sequences used for qRT-PCR. [file Table_1.docx]

Table S1. Primer sequences used for qRT-PCR

| Gen ID | Primer forward (5’ – 3’) | Primer reverse (5’ – 3’) |
| --- | --- | --- |
| PG21-1 | GCTATTCGTCACTCGGAGATTC | AATCCCAGTAGGCAGCTCCTTTG |
| PG14 | CAGGTCCTTGCAAACCAAATC | ATAGTTCCACCACCTTCCAC |
| PG69-1 | TTGAGTTGCAGCCATACTACCG | GAGTCTCCGTAAGCGTTGATAC |
| PG49 | GTATATGGAAGGTGCCAGGAAG | CATGGGAATGCTGTTTACTGC |
| PG23-3 | ATGCAAGGCGTCAGGATTTC | CGATTCCTCGACCATATGAC |
| PG46-3 | TAGCCACCTCACACTCTT | CGATTCCTCGACCATATGAC |
| PG17 | CAGACTTCGGCTGTTAAGATGG | GCCTTCACATGGTACGTCATTG |
| PG22-7 | CGGTGAATAGCCAGATGTTC | CCTGAGGCTGAAACCTTAAC |
| PG16 | GGCACCATCAATGGCAATGGCAAA | TAACCTCAAGCTTGCCACTCTCAG |
| ACT 7 | CGTTCTGTCCCTCTATGCCA | AGATCACGGCCAGCAAGATC |
| GAPDH | GTGGCTGTTAACGATCCCTT | GTGACTGGCTTCTCATCGAA |
